# Editorial: Insights in: health psychology

**DOI:** 10.3389/fpsyg.2025.1675009

**Published:** 2025-09-23

**Authors:** Changiz Mohiyeddini, Yingmin Zou, Dongjie Xie

**Affiliations:** ^1^Oakland University William Beaumont School of Medicine, Rochester, NY, United States; ^2^Sun Yat-sen University, Guangzhou, China; ^3^School of Special Education, Zhejiang Normal University, Hangzhou, China

**Keywords:** freedom, health psychology, mental health, psychosocial factors, behavioral health, public health interventions

## Introduction

Health psychology is a multidisciplinary field that explores the complex relationships between psychological factors and physical health (Taylor, 2017). This editorial addresses recent advancements in health psychology, offering insights from various studies to highlight the field's diverse and impactful nature.

In an era where the socio-economic foundations of societies are evolving faster than ever before, the interconnectedness of the global community has reached unprecedented levels. Technological advancements, economic shifts, and cultural exchanges are continually reshaping the way we live, work, and interact. These changes present both opportunities and challenges for individual and collective health and wellbeing (Marmot, 2005). As societies become more intertwined, the ripple effects of health-related issues can quickly transcend borders, affecting global populations, as was documented by the recent COVID-19 pandemic (Pfefferbaum and North, 2020). Health psychology, therefore, must adapt and respond to these dynamic changes by providing robust theoretical foundations and innovative applications aimed at protecting and enhancing individual and collective health and wellbeing (Glanz et al., 2015; Mohr et al., 2017). By advancing our understanding of how behaviors, emotions, and social factors influence health outcomes, health psychology can offer essential insights and interventions. This involves developing comprehensive models that consider the multifaceted nature of health in a globalized world and implementing evidence-based strategies that cater to diverse populations.

Innovative applications in health psychology, such as digital health interventions, community-based programs, and policy advocacy, are vital for mitigating the adverse effects of rapid societal changes. These initiatives can provide individuals with the necessary tools and resources to manage stress, build resilience, and maintain mental and physical health amid evolving circumstances. Moreover, health psychology must prioritize equity to ensure that advancements benefit all segments of society, particularly vulnerable and marginalized groups who may be disproportionately affected by socioeconomic shifts (Braveman and Gottlieb, 2014).

In conclusion, as societies continue to change and become more interconnected, health psychology must rise to the occasion by providing both theoretical insights and practical solutions. In doing so, health psychology can play a pivotal role in safeguarding the health and wellbeing of individuals and communities worldwide, ensuring that progress in one area does not come at the expense of another.

The contribution by Mohiyeddini to this Research Topic emphasized the importance of fundamental freedoms, such as freedom of speech, thought, and assembly, along with protection from discrimination, in maintaining psychological well-being. Restrictions on these freedoms have been linked to significant psychological distress, including anxiety, depression, and PTSD. The study underscores the need for comprehensive research to understand how different aspects of freedom interact to influence mental health outcomes.

Wang et al. underscored the ripple effect of life events in triggering non-suicidal self-injury (NSSI) among college students, which is mediated by sleep disturbances and psychotic-like experiences (PLEs). The strength of this study lies in its exploration of indirect psychological pathways, suggesting that interventions should go beyond surface-level stress management and directly address sleep quality and early psychotic symptoms. The article reinforces the growing call for more nuanced, system-level mental health interventions in academic settings.

Zeng et al. shifted the focus to maternal mental health and its delicate balance during childbirth. Their study identified the “Sense of Coherence” (SOC) as a critical buffer against psychological birth trauma. Notably, this protective effect is weakened or strengthened by other psychosocial elements such as fear, support, and childbirth readiness. This presents an actionable insight: cultivating psychological preparedness and structured support systems could play a decisive role in preventing trauma during one of life's most transformative moments.

Chen et al.'s meta-analysis drew our attention to a population that is often overlooked in suicide prevention efforts: cancer patients. Their work revealed a sobering reality—marital status, mental health history, pain, and even rural residence can significantly elevate suicide risk. The breadth and consistency of these findings demand an urgent expansion of psycho-oncological services, particularly in rural or under-resourced regions.

Concurrently, Ionescu et al. provided a detailed overview of patients with lumbar disc herniation, pointing to pain catastrophizing and unmet psychological needs as significant determinants of reduced quality of life. Post-surgical changes in catastrophizing, but not in unmet psychological needs, suggested that physical healing alone is insufficient. These findings advocate for integrative care that includes both somatic and psychological rehabilitation.

Aging populations were the subject of Cammisuli et al.'s observational study, which found that structured physical activity improves health-related quality of life in elderly women. Emotional wellbeing and fatigue levels improved significantly, especially in the context of reduced anxiety and depression. The implication is clear: exercise, often praised for its physical benefits, also constitutes a low-cost, high-impact strategy for improving mental health.

The psychological resources of adolescents were explored by Sun et al., who revealed that hope and psychological resilience are intimately linked, and together they shape mental health outcomes. This is not just theory—it is a clarion call for educators and policymakers to embed hope-building strategies and resilience training into school curricula.

Yoo et al. ventured into the neuroscience of pain by blending subjective reports with physiological indicators such as pupil dilation. Their work shows that past pain influences both perception and biological responses to current pain. In doing so, they challenge purely cognitive models of pain and hint at the rich interplay between memory, emotion, and the body.

Finally, Liu et al. investigated the impact of social support in online health communities. Their findings suggest that physician credibility and emotional resonance drive patient engagement, while excessive praise may dilute effectiveness. This has direct implications for how digital health platforms and providers should structure feedback and reputation systems.

## Conclusion

Taken together, these studies sketch a portrait of human vulnerability that is not rooted in pathology but in context: social, relational, developmental, and even digital. They also spotlight protective factors—hope, social support, and coherence—that can buffer against even the most adverse circumstances when nurtured. The future of mental healthcare must be as interdisciplinary and intersectional as the lives it aims to protect. These studies offer not only evidence, but also a roadmap.
